# Characteristics of Craniofacial Morphology and Occlusion in Shwachman–Diamond Syndrome: A Case Report of a Japanese Sibling Pair

**DOI:** 10.7759/cureus.53467

**Published:** 2024-02-02

**Authors:** Masahiro Takahashi, Masataka Ariwa, Tetsutaro Yamaguchi

**Affiliations:** 1 Department of Orthodontics, School of Dentistry, Kanagawa Dental University, Yokosuka, JPN

**Keywords:** dental features, cephalogram, craniofacial morphology, japanese sibling pair, shwachman–diamond syndrome

## Abstract

Shwachman-Diamond syndrome (SDS) is a rare autosomal recessive disorder mainly caused by mutations in the Shwachman-Bodian-Diamond syndrome gene on chromosome 7q11. Although skeletal abnormalities are a feature of SDS, no reports have focused on the craniofacial morphology of patients with SDS. Moreover, the detailed dental characteristics of SDS remain unknown. In the present case report, we evaluated the craniofacial morphology and dental findings of two patients with SDS. A Japanese adolescent sibling pair with SDS had the chief complaint of excessive overjet. Cephalometric analysis revealed similar craniofacial morphology in both patients: skeletal class I malocclusion with a hypodivergent pattern and labial inclination of the maxillary and mandibular incisors. A panoramic photograph showed the tendency of delayed permanent tooth eruption and replacement in both patients. These cases suggest that malocclusion requiring orthodontic treatment might be a feature of patients with SDS.

## Introduction

Shwachman-Diamond syndrome (SDS) (Online Mendelian Inheritance in Man (OMIM) #260400) is a rare autosomal recessive disorder mainly caused by mutations in the Shwachman-Bodian-Diamond syndrome (SBDS) gene on chromosome 7q11. The estimated incidence of SDS is 1/76,000, ranging from approximately 1/100,000 to 1/200,000 [1-3]. SDS more commonly affects male than female individuals [3]. In Japan, very few reports have described patients with SDS. SDS is usually characterized by exocrine pancreatic dysfunction, bone marrow failure, skeletal abnormalities (including metaphyseal chondrodysplasia, rib cage dysplasia, and osteopenia), short stature, and a variety of other less common features [4-7].

Some studies have shown that skeletal abnormalities are a feature of SDS [6,8-10]. Mäkitie et al. [10] reported that skeletal changes, as evaluated by radiography, were present in all 15 patients with SDS, and the severity and localization of these changes varied with age. Although some studies have focused on the skeletal abnormalities and phenotypes of patients with SDS, none have described the detailed features of the craniofacial morphology in these patients. Moreover, little is known about the dental characteristics of patients with SDS. Therefore, in the present case report, we describe the craniofacial morphology and dental features of a Japanese sibling pair with SDS.

## Case presentation

**Figure 1:**
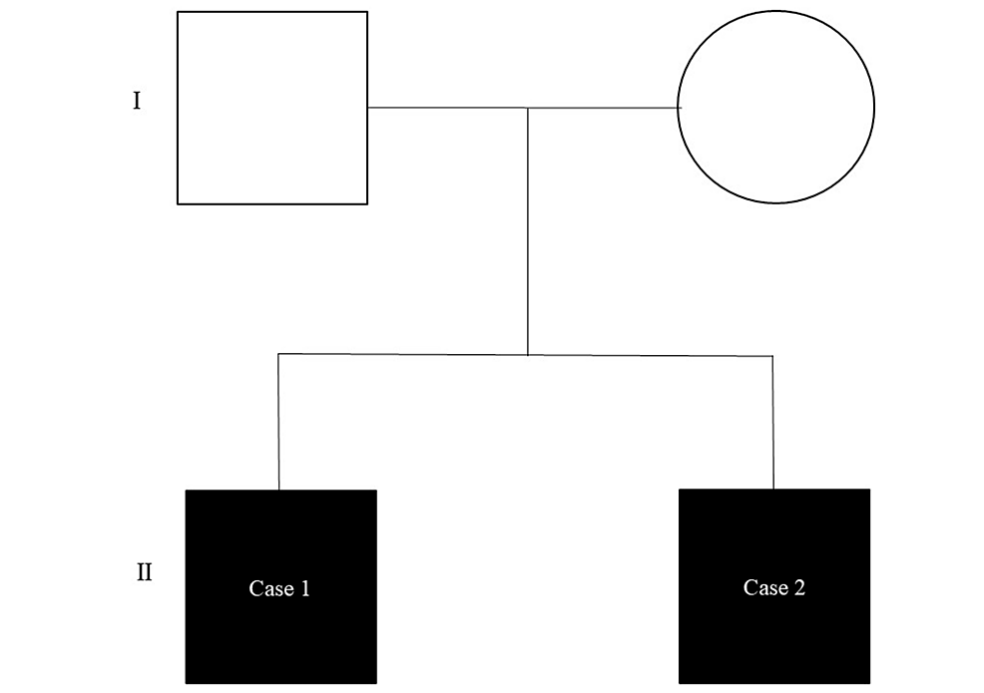
Pedigree of family including the two herein-described Japanese patients with SDS Squares and circle denote males and female, respectively. Filled symbols indicate affected individuals.

A Japanese sibling pair with SDS visited the Department of Orthodontics, Kanagawa Dental University Hospital when the older brother was 13 years old and the younger brother was 12 years old (Figure 1). Both patients with SDS presented with the chief complaint of excessive overjet.

Case 1

The older brother had been diagnosed with SDS at three months of age by confirmation of SBDS gene mutations. He had a medical history of pancreatic exocrine insufficiency, liver dysfunction, atrial septal defect, bronchial asthma, intellectual disability, and transfusion-dependent bone marrow failure. Moreover, he had undergone an unrelated bone marrow transplant (full human leukocyte antigen match) at the age of three years and 11 months.

**Figure 2:**
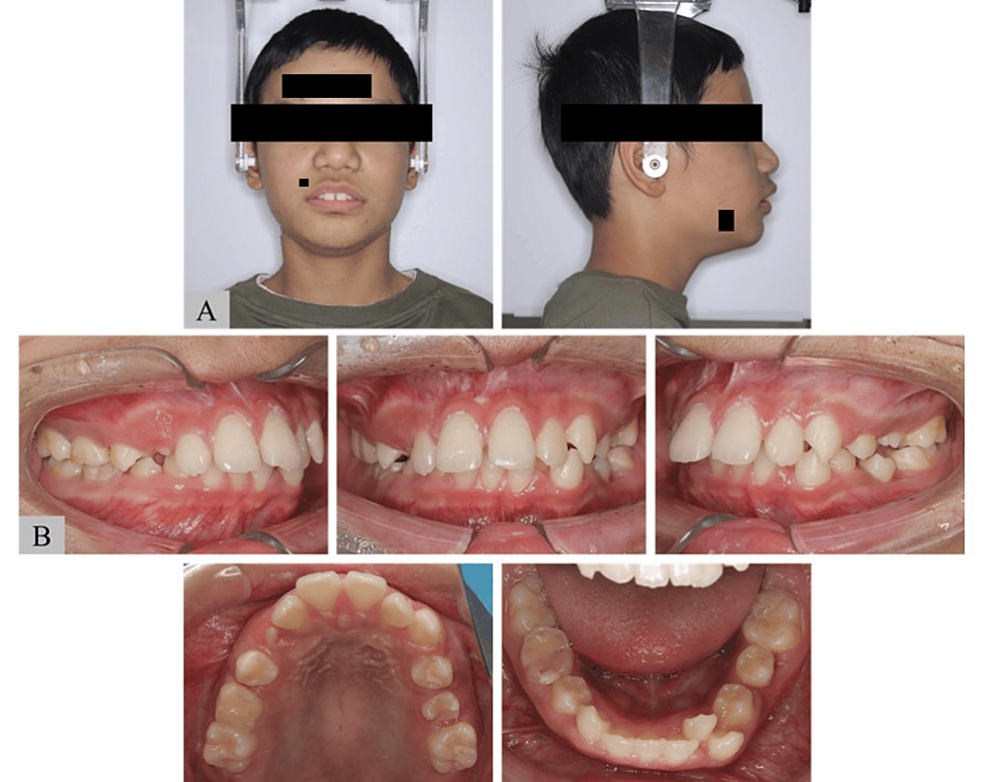
Photographs of Case 1 (A) Facial photograph and (B) intraoral photograph of older brother with Shwachman–Diamond Syndrome (SDS).

Upon presentation, his body height and weight were 137.9 cm and 31.6 kg, respectively, and he showed short stature. In the lateral facial view, the patient had a convex-type profile (Figure 2A). Intraorally, he had excessive overjet (+9.5 mm), deep overbite (+5.0 mm), and lower anterior crowding. His maxillary dental arch showed a V-shaped and spaced arch in the anterior part, and his mandibular dental arch showed moderate anterior crowding (Figure 2B). He also had a low tongue position and lip incompetence.

**Figure 3:**
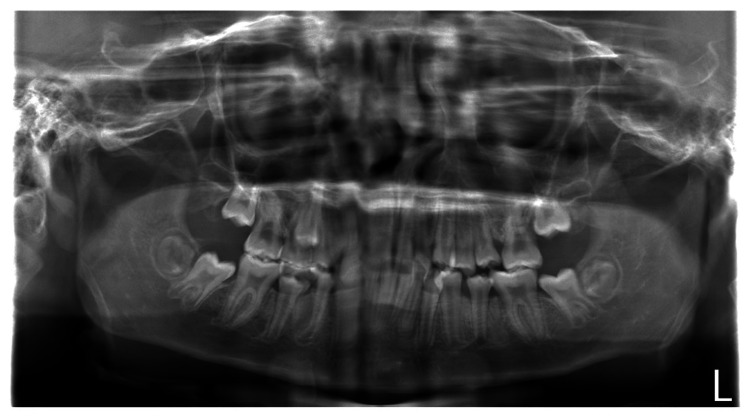
Panoramic radiograph of older brother with SDS SDS: Shwachman–Diamond Syndrome

The panoramic radiograph revealed a few primary tooth remnants and the presence of all third-molar tooth germs. There were no supernumerary or congenitally missing teeth (Figure 3). 

**Figure 4:**
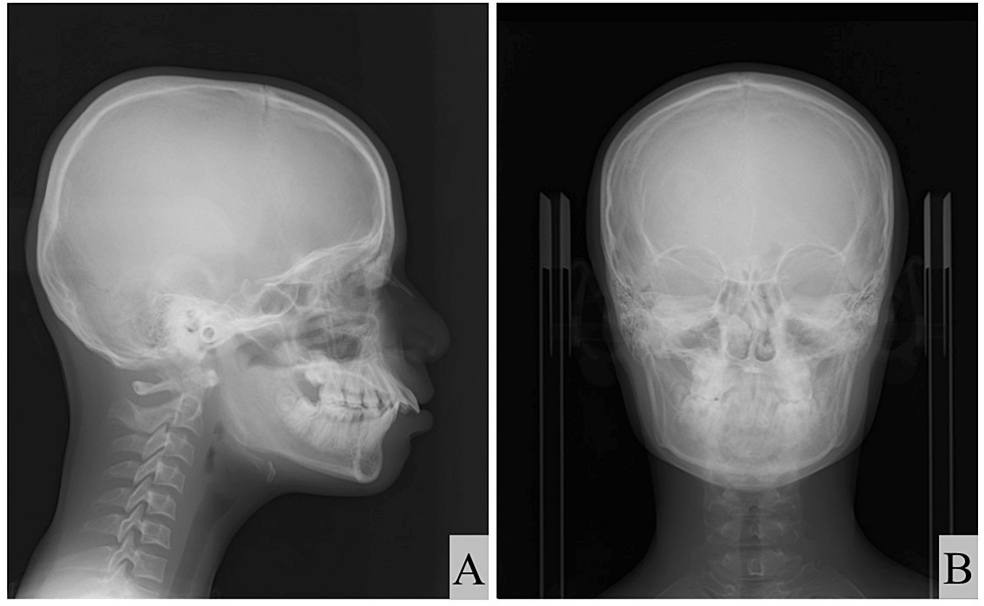
Cephalogram images of Case 1 (A) Lateral cephalogram and (B) posteroanterior cephalogram of older brother with Shwachman–Diamond Syndrome (SDS).

The lateral cephalometric analysis showed skeletal class I malocclusion (ANB; 3.6°) with a hypodivergent pattern (FMA; 21.6°) in the jaw relationship. Moreover, both the maxillary and mandibular incisors were proclined (U1-SN; 113.3°, IMPA; 104.3°) (Figure 4A and Table 1). The posteroanterior cephalogram showed no obvious deformity or deviation in the maxilla or mandible (Figure 4B). The posteroanterior cephalogram showed no obvious deformity or deviation in the maxilla or mandible (Figure 4B).

**Table 1:** Cephalometric analysis in Case 1 Norm: reference value, Initial: baseline value, SNA: Sella-Nasion-A, SNB: Sella-Nasion-B, ANB: A-point-Nasion-B-point, anteroposterior position between the maxilla and mandible, FMA: Frankfort mandibular angle, a cephalometric measurement utilized in cephalometric analysis to assess the vertical or horizontal facial growth pattern, IMPA: incisor mandibular plane angle, a cephalometric measurement utilized in cephalometric analysis to assess the inclination of the mandibular incisors relative to the mandibular plane, U1-SN: Upper incisor to Sella-Nasion, a cephalometric measurement utilized in cephalometric analysis to assess the inclination of the upper incisors in relation to the cranial base.

Measurement	Norm	Initial
Skeletal pattern		
SNA (°)	81.8	79.4
SNB (°)	78.6	75.8
ANB (°)	3.3	3.6
Convexity (°)	5.6	6.5
Facial angle (°)	85.1	83.4
Y-axis (°)	65.7	62.4
FMA (°)	26.3	21.6
Dental pattern		
IMPA (°)	94.7	104.3
U1-SN (°)	103.1	113.3

Case 2

The younger brother visited our department two years after his older brother. He had also been diagnosed with SDS at the age of one month by confirmation of mutations in the SBDS gene. Although he showed pancytopenia, low levels of exocrine pancreatic enzymes, steatorrhea, and intellectual disability, he was only taking medication for steatorrhea. Unlike his older brother, he had no bone marrow failure.

**Figure 5:**
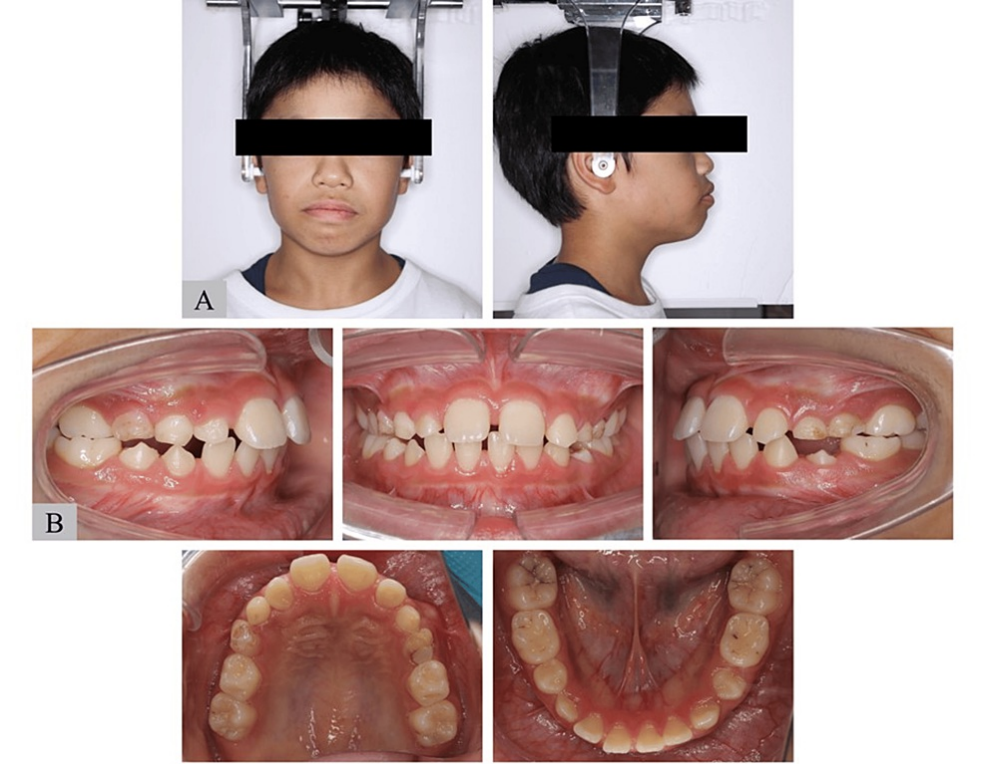
Photographs of Case 2 (A) Facial photograph and (B) intraoral photograph of the younger brother with Shwachman–Diamond Syndrome (SDS).

On presentation, the patient’s body height and weight were 133.6 cm and 29.4 kg, respectively, and he also showed short stature. In the lateral facial view, he had a convex-type profile (Figure 5A). Intraorally, his overjet and overbite were +5.0 and +3.0 mm, respectively. His maxillary dental arch showed a V-shaped and spaced arch in the anterior part. His facial profile, excessive overjet, and maxillary dental arch were similar to those of his older brother. By contrast, his mandibular dental arch showed a spaced arch in the anterior part, which differed from the features of his brother (Figure 5B). He also had a low tongue position.

**Figure 6:**
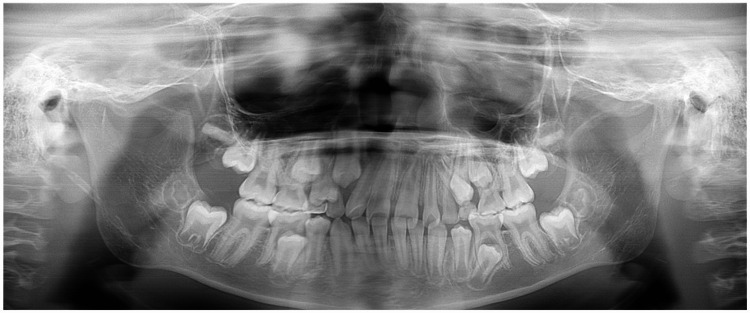
Panoramic radiograph of the younger brother with SDS The panoramic radiograph showed an ectopic position of the premolars on the left side within the alveolar bone in both the maxilla and mandible. SDS: Shwachman–Diamond Syndrome

The panoramic radiograph findings were also similar to those of his older brother in several ways (i.e., the presence of all third molar tooth germs and the absence of supernumerary and congenitally missing teeth). However, the younger brother showed an ectopic position of the premolars within the alveolar bone and delayed permanent tooth eruption and replacement (Figure 6). 

**Figure 7:**
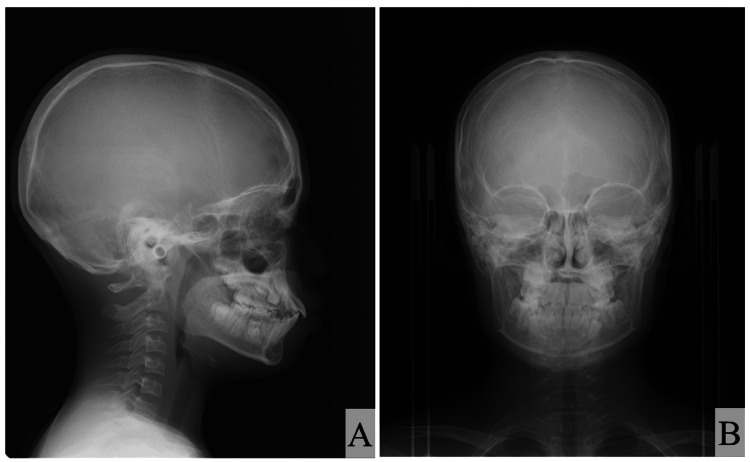
Cephalogram images of Case 2 (A) Lateral cephalogram and (B) posteroanterior cephalogram of the younger brother with Shwachman–Diamond Syndrome (SDS).

The lateral cephalometric analysis showed similar characteristics to those of his older brother (i.e., skeletal class I malocclusion (ANB; 3.9°) with a hypodivergent pattern (FMA; 19.1°) and labial inclination of the maxillary and mandibular incisors (U1-SN; 122.6°, IMPA; 105.1°)] (Figure 7A and Table 2). He also had no obvious deformity or deviation in the maxilla or mandible (Figure 7B). He also had no obvious deformity or deviation in the maxilla or mandible (Figure 7B).

**Table 2:** Cephalometric analysis in Case 2 Norm: reference value, Initial: baseline value, SNA: Sella-Nasion-A, SNB: Sella-Nasion-B, ANB: A-point-Nasion-B-point, anteroposterior position between the maxilla and mandible, FMA: Frankfort mandibular angle, a cephalometric measurement utilized in cephalometric analysis to assess the vertical or horizontal facial growth pattern, IMPA: incisor mandibular plane angle, a cephalometric measurement utilized in cephalometric analysis to assess the inclination of the mandibular incisors relative to the mandibular plane, U1-SN: Upper incisor to Sella-Nasion, a cephalometric measurement utilized in cephalometric analysis to assess the inclination of the upper incisors in relation to the cranial base.

Measurement	Norm	Initial
Skeletal pattern		
SNA (°)	81.8	84.9
SNB (°)	78.6	81.0
ANB (°)	3.3	3.9
Convexity (°)	5.6	7.1
Facial angle (°)	85.1	86.6
Y-axis (°)	65.7	59.8
FMA (°)	26.3	19.1
Dental pattern		
IMPA (°)	94.7	105.1
U1-SN (°)	103.1	122.6

## Discussion

The incidence of SDS is very low. Patients with SDS have various disorders, such as exocrine pancreatic dysfunction, bone marrow failure, skeletal abnormalities, and short stature [4-7]. However, because patients with SDS rarely visit orthodontic departments, their craniofacial morphology and dental features have remained largely unknown. To the best of our knowledge, this is the first case report to describe the craniofacial morphology and dental features of a Japanese adolescent sibling pair with SDS.

The cephalometric analysis revealed that both patients with SDS showed similar craniofacial morphology: skeletal class I malocclusion with a hypodivergent pattern and labial inclination of both the maxillary and mandibular incisors. Mäkitie et al. [10] reported that five patients with SDS showed a normal skull shape with reduced thickness of the cortical bone in the cranium on lateral skull radiographs. Moreover, three patients with SDS showed Wormian bones in the cranium [10]. However, the lateral cephalometric radiographs in our two patients did not show the reduced thickness of the cortical bone or Wormian bones in the cranium. Although it is difficult to discuss and compare the different findings of craniofacial morphology because of the low numbers of such studies and/or case reports, one possible explanation for these results relates to the catching up of bone maturation with growth. The panoramic radiographs and intraoral photographs in both our patients with SDS indicated that the permanent teeth in the lateral tooth group tended to erupt and be replaced slowly. Moreover, an ectopic position of the premolars within the alveolar bone was observed in the younger brother. The differences in bone maturation between different patients with SDS may explain these findings. However, the findings were from only two cases, which is a limitation of this case report. Further studies and case reports are needed to examine the craniofacial morphology, delayed permanent tooth eruption, and ectopic tooth positions within the alveolar bone in more detail.

It is important to share the characteristics of craniofacial morphology and dental findings in patients with rare congenital diseases associated with malocclusion, such as SDS. Although orthodontic perspectives in patients with SDS have not been reported previously, we believe it is crucial to verify the presence of malocclusion in these patients. If malocclusion is identified in SDS patients, we should consider interdisciplinary treatment, including orthodontic intervention, to be of significance. The continued accumulation of information regarding craniofacial morphology and dental findings in patients with SDS will contribute to the establishment of orthodontic treatment for these patients.

## Conclusions

Cephalometric analysis revealed that a Japanese sibling pair with SDS had skeletal class I malocclusion with a hypodivergent pattern, labial inclination of both the maxillary and mandibular incisors, and excessive overjet. Moreover, the panoramic radiographs and intraoral photographs indicated that the permanent teeth in the lateral tooth group tended to erupt and be replaced slowly. Both patients with SDS also showed short stature. These cases suggest that some patients with SDS might have malocclusion requiring orthodontic treatment.
